# Familial Glucocorticoid Deficiency: the changing landscape of an eponymous syndrome

**DOI:** 10.3389/fendo.2023.1268345

**Published:** 2023-12-21

**Authors:** Avinaash V. Maharaj

**Affiliations:** Centre for Endocrinology, William Harvey Research Institute, Queen Mary University of London (QMUL), London, United Kingdom

**Keywords:** adrenocorticotropin, steroidogenesis, hypocortisolaemia, zona fasciculata, multi-systemic

## Abstract

Familial Glucocorticoid Deficiency encompasses a broad spectrum of monogenic recessive disorders that theoretically solely abrogate cortisol biosynthesis. In reality, delineating clear genotype-phenotype correlations in this disorder is made complicated by marked phenotypic heterogeneity even within kindreds harbouring identical variants. Phenotypes range from isolated glucocorticoid insufficiency to cortisol deficiency plus a variety of superimposed features including salt-wasting and hypoaldosteronism, primary hypothyroidism, hypogonadism and growth defects. Furthermore, mutation type, domain topology and perceived enzyme activity do not always predict disease severity. Given the high burden of disease and implications of a positive diagnosis, genetic testing is crucial in the management of patients warranting detailed delineation of genomic variants including viable functional studies.

## 1 Introduction

### 1.1 Historical origins of Familial Glucocorticoid Deficiency

Unsubstantiated reports of Addisonian-like familial disease associated with hyperpigmentation were recorded from as early as 1900 but the disorder first came to prominence in 1959 when Shepard and colleagues described a unique form of hypocortisolaemia in two siblings with preserved mineralocorticoid function (1). Over subsequent decades, the phenotypic spectrum evolved to include a distinct constellation of features including hyperpigmentation, recurrent hypoglycaemia, seizures, high plasma deoxycorticosterone and tall stature (2–9). Initially designated as hereditary adrenocortical unresponsiveness to adrenocorticotropin (ACTH) (4), this syndrome was eventually termed Familial Glucocorticoid Deficiency (FGD).

### 1.2 Physiological regulation of cortisol production

**Figure 1:**
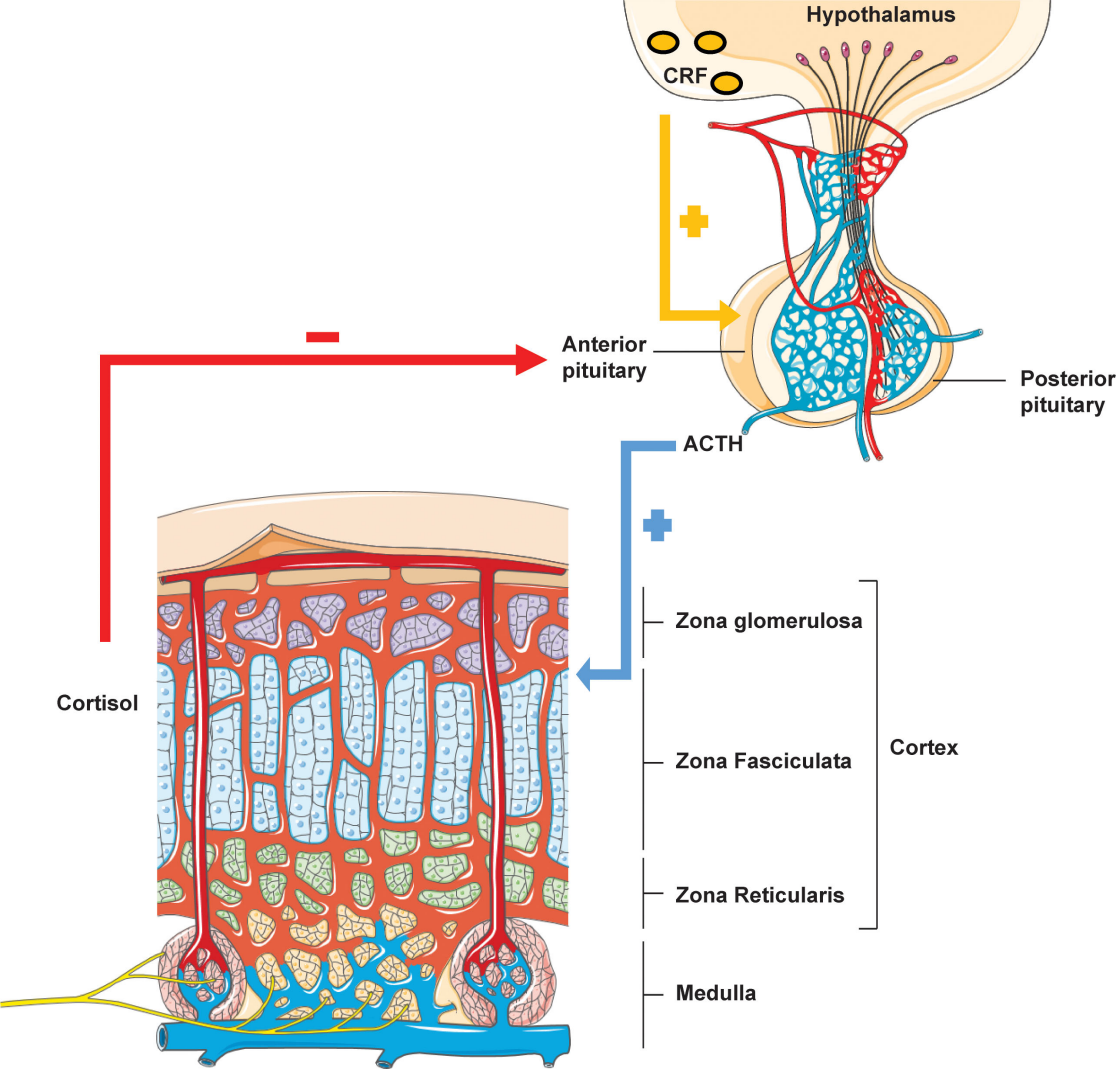
Hypothalamic-pituitary-adrenal axis. Rhythmicity of the HPA axis is driven by hypothalamic tropic factor CRH which stimulates anterior pituitary ACTH production. ACTH acts on the adrenal zona fasciculata to mediate glucocorticoid biosynthesis. Cortisol itself then exerts feedback inhibition of both ACTH and CRH via a negative feedback loop.

Higher brain centres regulate the synthesis of endogenous cortisol through an intricate negative feedback pathway governed by the tropic Corticotrophin releasing factor (CRF) secreted by the hypothalamo-adenohypophyseal portal system (**Figure 1**). CRF preferentially binds to the CRF type 1 G-protein coupled receptor (CRF-1R) and acts as a secretagogue to potentiate ACTH release from the anterior pituitary. ACTH then acts as an agonist for the melanocortin 2 receptor (MC2R), expressed in adrenocortical cells of the adrenal zona fasciculata, thereby producing cortisol via modulation of adenylate cyclase/protein kinase A signalling. Cortisol mediates the negative feedback loop via the centrally expressed glucocorticoid receptor. Glucocorticoid mediated feedback may be divided into: (i) non-genomic rapid inhibition of glutamate release at the hypothalamic paraventricular nucleus via endocannabinoid synthesis, (ii) genomic prefrontal limbic regulation and, (iii) destabilization of hypothalamic pituitary adrenocortical axis-activating neuropeptide mRNA (10). Loss of feedback regulation forms the basis of ACTH resistance syndromes of which defects in *MC2R* were the first to be elucidated.

## 2 Familial glucocorticoid deficiency (GCCD1/FGD Type 1) – melanocortin-2 receptor (*MC2R*) defects (OMIM #202200)

**Figure 2:**
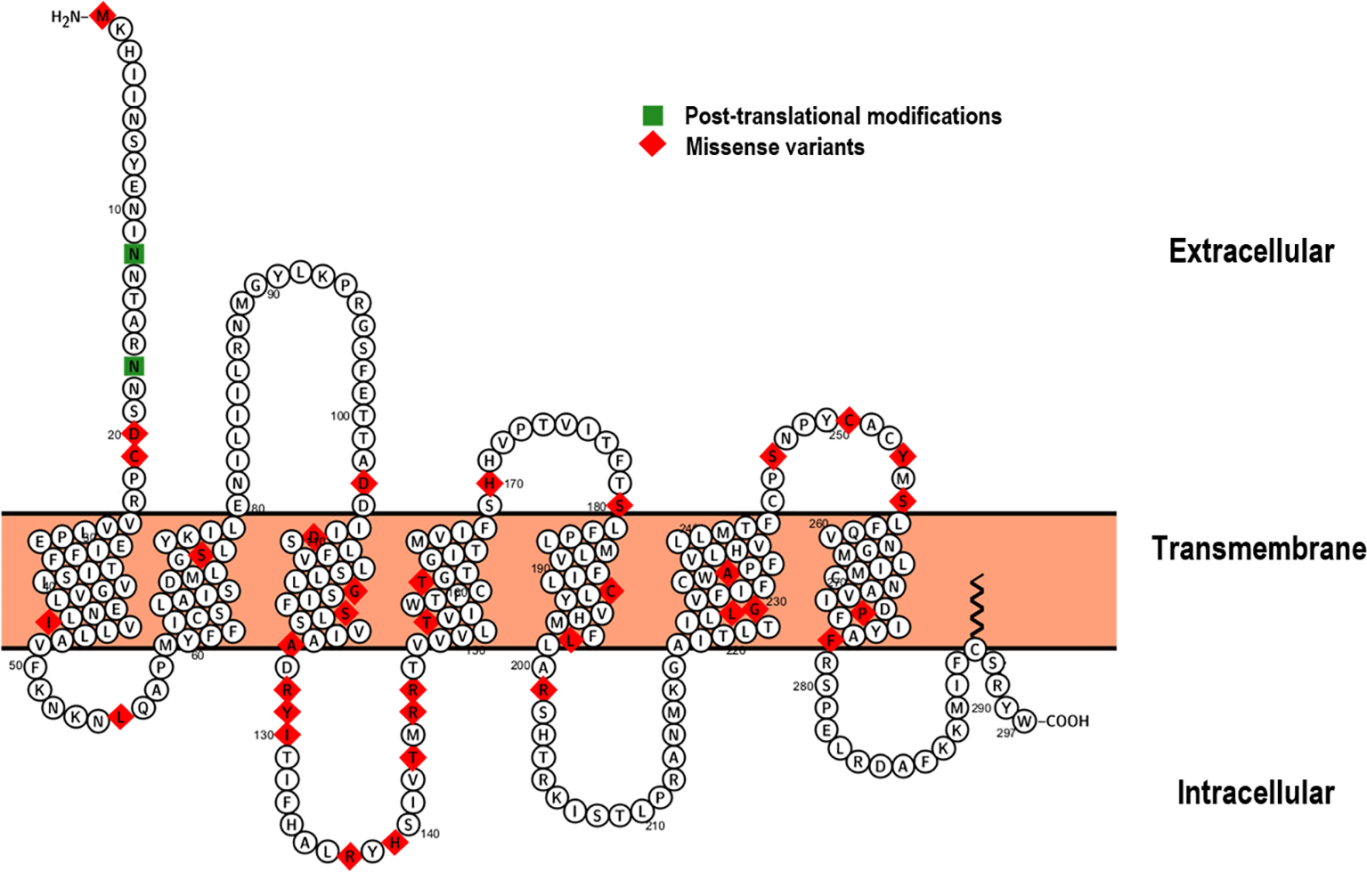
Proteoform structure of the ACTH receptor (15). The topological (extracellular, transmembrane and cytoplasmic) domains are outlined and sites of missense variation highlighted (red). The majority of *MC2R* variants causing FGD Type 1 are associated with defective receptor trafficking and mainly occur within the transmembrane domains (16). Sites of post-translational modifications are indicated in green.

The ACTH receptor is a seven transmembrane domain receptor encoded by the *MC2R* gene, which maps onto the short arm of chromosome 18 and consists of 2 exons, the first of which is non-coding/untranslated whilst the latter encodes the full sequence of the receptor. Following isolation and sub-cloning of the human ACTH receptor in 1992 (11), the first reported kindreds with primary adrenal insufficiency secondary to defects in *MC2R* were characterised by Clark et al. and Tsigos et al. in 1993 (12, 13). Since these initial reports, around 48 mutations have been described in association with FGD (Human Gene Mutation Database http://www.hgmd.cf.ac.uk) (14), the majority of which are missense/nonsense variants (**Figure 2**) in addition to small genomic deletions and insertions. Variants in *MC2R* account for about 25% of FGD cases, the majority of which are non-conservative, single amino acid substitutions that likely abrogate cyclic adenosine monophosphate (cAMP) generation and impair receptor trafficking (17–19).

Classically in FGD Type 1, patients are mineralocorticoid replete however, in clinical practice, the picture may be less unambiguous. Several reports have alluded to mild disruptions of the renin-angiotensin-aldosterone axis in patients with *MC2R* defects, in whom early glucocorticoid replacement may mask aldosterone insufficiency (20, 21). Furthermore, several patients with homozygous frameshift truncating variants have presented in the neonatal period with transient salt wasting (20). Interestingly *Mc2r^-/-^* mice demonstrate reduced serum aldosterone levels (22). This disparity may reflect the fact that the majority of human subjects harbour missense mutations that retain some degree of enzyme activity. Patients also invariably present with early and significant hyperpigmentation due to the action of markedly increased ACTH on the Melanocortin 1 receptor (*MC1R*). This association was clearly demonstrable in a patient with FGD harbouring homozygous missense variants in both *MC2R* and *MC1R* and lacking the ‘classic’ hyperpigmented phenotype (23).

An often overlooked but increasingly recognised feature of FGD Type 1 is hypothyroidism (18, 24–29). Patient phenotypes range from transient neonatal hypothyroidism that normalise with short term thyroxine replacement to subclinical hypothyroidism and persistent thyroid hypo-function (28, 29). Despite assertions that elevated ACTH levels may inhibit thyroid stimulating hormone release (30), the exact mechanism underlying the incidence of hypothyroidism in patients with FGD remain to be elucidated. Interestingly, another feature recognised in patients with ACTH receptor defects is reduced adrenal androgen production or lack of adrenarche. Weber et al. demonstrated consistently sub-optimal serum DHEAS levels in 6 patients with *MC2R* variants (31). This model of ‘functional’ ACTH deficiency suggests that ACTH at least partially regulates adrenarche given that patients with central ACTH deficiency (hypopituitarism) also exhibit low levels of adrenal androgens (31–34).

Tall stature is an inconsistent but historically common feature of FGD type 1 despite an unaffected Growth hormone-IGF-1 axis (35). Although the exact mechanism underlying this phenotype is unclear, it is likely theorised to be due to the sustained effect of markedly elevated Adrenocorticotropin levels on the growth plate (35, 36). This also correlates to a later median age at diagnosis (2.0 years) when compared to FGD Type 2 (37). ACTH has been shown *in vitro* to increase rat chondrocyte progenitor cell proliferation and matrix production (38). Interestingly, growth trajectories return to normal once glucocorticoid treatment is instituted. *Mc2r* knockout mice do not recapitulate this trait and demonstrate similar body lengths to wild-type littermates (22). There is however little genotype phenotype correlation and disease severity and onset is highly variable (39).

## 3 Familial glucocorticoid deficiency (GCCD2/FGD Type 2) – melanocortin-2 receptor accessory protein (*MRAP*) (OMIM #607398)

**Figure 3:**
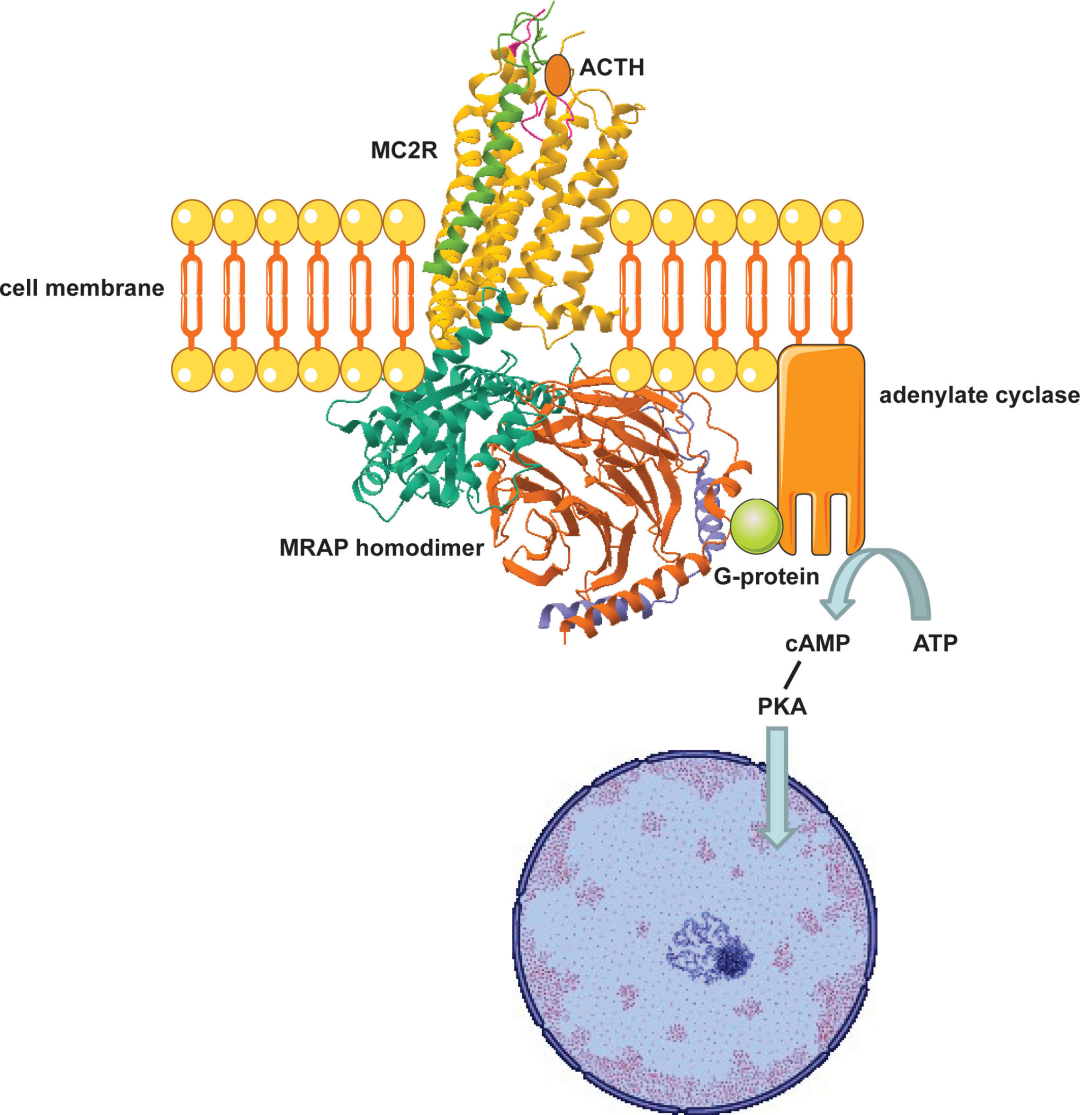
Adrenocortical cell surface MC2R-MRAP complex mediates glucocorticoid biosynthesis. MRAP traffics MC2R to the cell surface where ligand binding (ACTH) induces a G-protein coupled increase in cAMP which phosphorylates Protein Kinase A (PKA). PKA then induces transcription of specific intra-nuclear factors that mobilise STAR activity and expression of enzymes involved in steroidogenesis.

After initial characterisation of loss of function defects in *MC2R* as a primary cause of FGD, it became apparent that other genetic factors were involved in pathogenesis of FGD given the existence of several families with an isolated adrenal insufficiency phenotype and negative *MC2R* genomic screening. *In vitro* expression studies by Noon et al. revealed that a fluorescent tagged *MC2R* cDNA clone was able to traffic to the cell membranes of adrenocorticotropin resistant murine adrenocortical tumour cells but remained restricted to the endoplasmic reticulum in non-adrenal cells. This suggested that an adrenal derived co-factor was necessary for MC2R expression (40, 41) (**Figure 3**).

In 2005, Metherell et al. conducted autozygosity mapping in a single family in which runs of homozygosity identified a 2.2 Mbp region. Within this region, the adrenally expressed chromosome 21 open reading frame 61 (*C21orf61*) gene previously encoding a fat-associated low-molecular-weight protein, was identified. Variants in this gene, retitled the melanocortin 2 receptor accessory protein (*MRAP*), were subsequently identified in several families with FGD (42). Variants in *MRAP* (FGD Type 2) account for 20-25% of FGD cases, with around 15 mutations being described since initial characterisation of this gene (37, 42–45). The majority of variants are splice site/nonsense that ultimately lead to a truncated and non-functional receptor. Patients generally present at an earlier age (median age of 0.08 years at diagnosis) with normal stature when compared to subjects with *MC2R* variants (37, 45, 46). This suggests reduced exposure to the unfettered actions of ACTH on the growth plate seen in FGD Type 1. *Mrap^-/-^* mice phenocopy the isolated glucocorticoid deficiency and normal mineralocorticoid function of human subjects. Interestingly, the adrenals from knockout mice are small with indistinct cortical zonation and dysregulated accumulation of WNT4/β-catenin (47).

## 4 Partial loss of function mutations in steroidogenic acute regulatory protein (*STAR*) and cytochrome P450 side chain cleavage enzyme (*CYP11A1*)

### 4.1 *STAR* (GCCD3, OMIM #609197)

**Figure 4:**
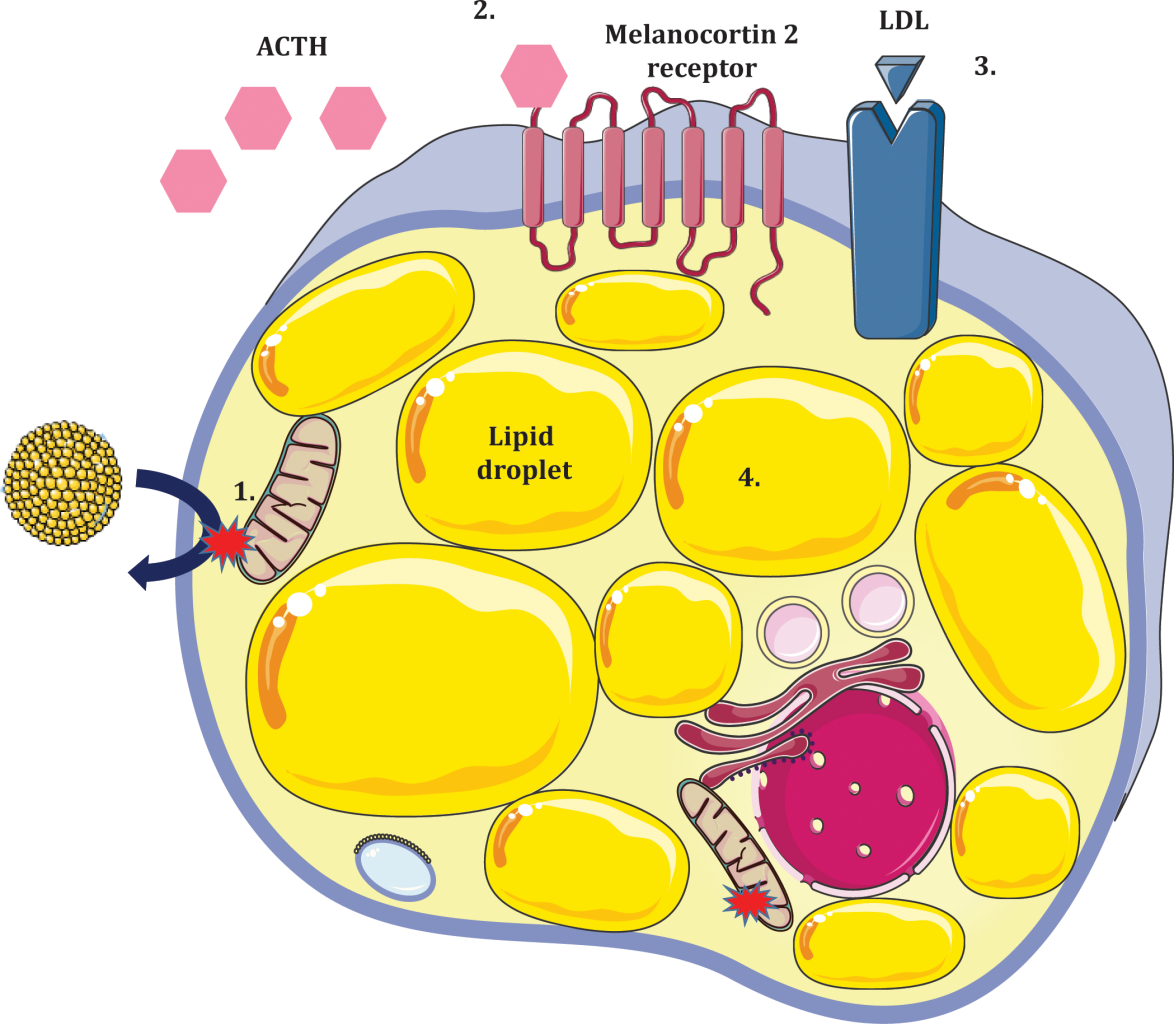
Two ‘hit’ model of lipoid congenital adrenal hyperplasia due to deficiency of STAR in adrenocortical cells. 1. Absence of STAR prevents cholesterol import to the inner mitochondrial membrane (first hit). Some residual STAR-independent steroidogenesis occurs but relative hypocortisolaemia ensues. 2. Loss of negative feedback to the anterior pituitary results in increased ACTH production. 3. Steroidogenic stimuli promote expressivity of LDL receptors which increase adrenocortical cholesterol uptake. 4. Accumulation of intracellular lipids causes oxidative damage and cellular stress (second hit) further impeding steroidogenic potential leading to hyperplastic adrenals.

Defects in *STAR* disrupt steroidogenesis globally resulting in classic congenital lipoid adrenal hyperplasia (CLAH). Patients invariably present with hyper-reninaemic hypoaldosteronism in the setting of hypocortisolaemia, enlarged adrenals due to progressive lipid deposition and gonadal insufficiency. A two hit model has been proposed to account for pathogenesis of CLAH (**Figure 4**); the first ‘hit’ being lack of STAR and the second being cholesterol mediated oxidative damage. In STAR deficient adrenocortical and testicular Leydig cells, steroidogenesis is markedly diminished with the exception of minimal residual STAR-independent steroid biosynthesis. The primary biosynthetic defect leads to compensatory increases in ACTH and LH which promote LDL receptor mediated cholesterol uptake and *de novo* synthesis. Progressive lipid accumulation leads to mitochondrial oxidative damage and cellular stress ultimately abolishing residual STAR-independent steroidogenic capacity (48, 49). Early fetal destruction of Leydig cell integrity in 46,XY subjects leads to lack of testosterone and feminized external genitalia (50). The fetal ovary, on the contrary, is relatively preserved until puberty when gonadotrophin stimulation leads to cholesterol accumulation.

In some patients, mutations in *STAR* are associated with retention of up to 20% of wild type activity and a mild phenotype. These partial loss of function variants result in non-classic CLAH (NCLAH), often indistinguishable from FGD due to an isolated adrenal phenotype (51–55). Age at presentation is highly variable, ranging from 18 months to adulthood (51, 56). NCLAH cortisol deficient patients exhibit normal external genitalia and consonant pubertal development however, primary gonadal failure may occur progressively over time (51, 53).

Expression of a mutant Star protein (N47-StAR) in knockout mice (*Star* ^−/−^) gave rise to a partial loss of function phenotype. When compared to wild type transgenic mice, *Star* ^−/−N47Tg^ mice had lower basal and stimulated corticosterone levels. *Star* ^−/−N47Tg^ mice had normal external genitalia but exhibited progressive gonadal insufficiency with aging (57).

### 4.2 Cholesterol side-chain cleavage enzyme, P450scc (*CYP11A1*) (OMIM #613743)

*CYP11A1*, a mitochondrial monooxygenase regulates placental production of progesterone required for maintenance of gestational viability (48, 58). Absolute deficiency of this enzyme was previously thought to be incompatible with term gestation i.e. embryonically lethal, however, several patients with side chain cleavage enzyme deficiency have been reported. It is postulated that in these instances, persistence of the corpus luteum throughout pregnancy compensates for the placentation defect (59). Severe loss of function mutations in *CYP11A1* produce a phenotype not dissimilar to that of CLAH due to *STAR* deficiency. Primary adrenal failure in combination with 46, XY sex reversal characterise these patients although unlike *STAR* deficient subjects, adrenal size is unaffected.

However, like *STAR*, partial loss of function mutations can produce a phenotype consistent with non-classic CLAH (60). Furthermore, partial inactivating mutations in *CYP11A1* have been implicated in development of an isolated glucocorticoid deficiency. Parajes et al. (2011) described a homozygous mutation (p.R451W) in two 46, XY male siblings who presented with primary adrenal insufficiency and normal external genitalia without hypogonadism (61). Unlike severe enzyme deficiency which presents within the first 10 days of life, partial loss of function variants in *CYP11A1* are associated with later ages of onset ranging from 6 months to 15 years (62–65).

Maharaj et al. (64) described a heterozygous missense variant (rs6161, c.940G>A, p.Glu314Lys) in *CYP11A1*, previously designated benign, in a cohort of 19 probands (13 families) with isolated glucocorticoid deficiency. The variant occurred *in compound heterozygosity* with a second gene disrupting allelic hit in *CYP11A1* in 17 probands and in combination with two synonymous single nucleotide variants (p.Thr330= and p.Ser391=) in two probands. *In vitro* splicing assays demonstrated exon skipping due to aberrant splicing for these three variants. The impact of the rs6161 variant on splicing was further corroborated by several subsequent groups (66–69).

*Cyp11a1* ^-/-^ mice do not survive weaning and demonstrate marked corticosterone and aldosterone deficiency when compared to wild-type litter mates. Interestingly, corticosteroid administration prolonged survival until adulthood. *Cyp11a1* ^-/-^ XY males exhibit sex reversal with feminization of external genitalia and disorganization of internal genitalia. Similar to human counterparts, knockout adrenals are small however they demonstrate progressive lipid droplet accumulation (70).

## 5 FGD-like syndrome due to altered cellular redox status (*NNT*, *TXNRD2*)

### 5.1 Nicotinamide nucleotide transhydrogenase (*NNT*) (GCCD4, OMIM #614736)

Single nucleotide polymorphism genotyping and targeted exome sequencing of consanguineous kindreds with adrenal insufficiency identified variants in the gene encoding the inner mitochondrial membrane enzyme, nicotinamide nucleotide transhydrogenase (*NNT*) (71). The initial 15 probands characterised by Meimaridou et al. presented with a classical FGD phenotype indistinguishable from mutations in *MC2R* and *MRAP*. Patients were diagnosed before age 40 months with biochemical evidence of marked hypocortisolaemia, elevated ACTH and normal renin and aldosterone levels. A significant number of patients (8 out of 15) presented with sequelae of hypoglycaemia. A further study noted that some patients were mineralocorticoid deficient highlighting a degree of phenotypic variability (46, 72).

Lentiviral knockdown of *NNT* in an H295R (adrenocortical cancer) cell line demonstrated increased mitochondrial reactive oxygen species generation in the knockdown condition with a lowered reduced glutathione (GSH) to oxidized glutathione (GSSG) ratio. This was indicative of an altered cellular redox balance with subsequent perturbations in adrenal steroidogenesis (71, 73). A reduced GSH : GSSG ratio has also been seen in other *in vitro* models of adrenal disease where oxidative stress is implicated in the pathogenic mechanism, including *TXNRD2* and *AAAS*-KD H295R adrenocortical cell lines (74, 75). Similarly, metabolic profiling of glucocorticoid deficient *fdx1b* ^−/−^ zebrafish revealed significant alterations to glutathione metabolism and biochemically lowered GSH to GSSG ratios in ferredoxin null zebrafish larvae (76).

Heterozygous loss-of-function *NNT* mutations have been linked to left ventricular non-compaction (LVNC) in two probands and their families. *Nnt* deficient zebrafish cardiomyocytes demonstrated reduced proliferation and contractility leading to cardiac oedema. *In vivo*, co-injection of wild-type human *NNT* mRNA was able to rescue the cardiac oedema phenotype whilst mutagenized human *NNT* ORF constructs failed to rescue morpholinos-induced cardiac dysfunction (77). Roucher-Boulez et al. subsequently identified a novel homozygous *NNT* mutation (p.R379*) in a single patient who exhibited features of glucocorticoid insufficiency with progressive left ventricular hypertrophy (78).

C57BL/6J mice possess an intrinsic inactivating mutation in *Nnt* resulting in an untranslated protein. Interestingly, C57BL/6J *Nnt*-null mice do not develop cardiomyopathy (79) but they do have a lower adrenal reserve with attenuated corticosterone levels, both basally and following ACTH provocation. Adrenal histology revealed a markedly disorganized zona fasciculata with increased apoptosis (71). Mitochondria isolated from *Nnt* null mice exhibited an increased oxidized/reduced glutathione ratio and impaired ability to metabolize organic peroxide suggesting that loss of *Nnt* leads to redox imbalance (80). At protein level, *Nnt* null mice exhibited a 65% reduction in expression of *Cyp11a1*, which catalyses the rate limiting step of steroidogenesis (73).

### 5.2 Thioredoxin reductase 2 (*TXNRD2*) (GCCD5, OMIM #617825)

Amongst the genetic causes of FGD, *TXNRD2* is perhaps the most enigmatic. Until recently, only one consanguineous kindred was known to harbour a deleterious homozygous variant in *TXNRD2* in association with a phenotype of glucocorticoid insufficiency. In 2014, Prasad et al. characterised 7 individuals from a Kashmiri kindred who were found to have a stop gain variant, p.Y447*, associated with loss of TXNRD2 (74, 81). The age of presentation was highly variable ranging from 0.1 to 10.8 years (74). Of the glucocorticoid deficient family members genotyped, only one had co-morbid heart defects precipitating cardiac failure; cardiomyopathy being a recognised feature of *TXNDR2* haploinsufficiency (82).

Sibbing et al. identified two heterozygous mutations in *TXNRD2* (p.G375R and p.A59T) in 3 individuals with dilated cardiomyopathy (DCM). When cell survival was used as a marker of *Txnrd2* function, neither mutant construct was able to rescue *Txnrd2* function in *Txnrd2* ^-/-^ GSH depleted mouse embryonic fibroblasts in contrast to a wild type construct (83).

In 2022, a new study highlighted a novel homozygous missense variant in *TXNRD2* (c.1081G>A, p.V361M) in a proband with glucocorticoid and gonadal insufficiency but normal cardiac function (84). This adds credence to the impact of TXNRD2 as a player in the pathogenesis of isolated glucocorticoid deficiency and further broadens the phenotypic continuum of this disorder.

## 6 FGD-like syndrome due to defective DNA replication (Minichromosome maintenance 4, *MCM4*) (OMIM #609981)

In 2012, dual reports of partial *MCM4* deficiency in 14 probands from the Irish Traveller population highlighted a unique syndrome that encompassed adrenal insufficiency, intrauterine and postnatal growth restriction, microcephaly and natural killer cell (NK) deficiency (85, 86). The variant highlighted (and to date, the only variant associated with this disorder) was a homozygous splice site substitution c.71-2A>G leading to a single base cDNA insertion c.70_71insG and frameshift truncation, p.P24Rfs*4. Unlike other forms of FGD, the adrenal phenotype was relatively mild with onset of adrenal insufficiency often in late childhood. In healthy control derived peripheral lymphocytes, two MCM4 isoforms are detectable at 96KDa and 85KDa. In patient cells, only the minor isoform is present. This smaller protein is touted to lack the N-terminal MCM4 domain and may partially rescue patient phenotype (85, 86).

Complete loss of *Mcm4* in mice is lethal. Hypomorphic *Mcm4* ^Chaos3/–Mcm3+/–^ mice are viable and demonstrate abnormal adrenal morphology. The adrenal capsule is thinned with significant numbers of non-steroidogenic *Cyp11a1*/*Cyp11b1* negative cells that are on the contrary, Gata-4 and Gli1-positive. This remodelled adrenal cortex demonstrates a lack of steroidogenic output in keeping with human disease (85, 87).

## 7 Disorder of sphingolipid metabolism due to SGPL1 deficiency (OMIM #617575) leading to primary adrenal insufficiency and steroid resistant nephrotic syndrome

Until recently, inborn errors of sphingolipid metabolism due to single enzyme defects within the sphingolipid pathway have been characterised by their predominantly neurological phenotype. Sphingosine-1 phosphate lyase insufficiency syndrome (SPLIS) due to defects in *SGPL1*, which coordinates the final degradative step in the sphingolipid pathway, is a newly described multi-systemic disorder, in which adrenal failure features prominently (88–90).

Sphingolipid synthesis involves a series of tightly regulated, enzyme-catalysed steps that initiate in the endoplasmic reticulum from non-sphingolipid precursors to biosynthesis of higher order complex glycosphingolipids within the Golgi apparatus. Despite the diversity within the biosynthetic pathway, sphingolipid metabolism begins with a common entry point and exit via a single degradative pathway. This common initial step involves the coupling of cytosolic serine and palmitoyl CoA to 3-ketodihydrosphingosine through the action of serine palmitoyltransferase (*SPT*) whilst *SGPL1* executes the penultimate step of the metabolic pathway, catalytic cleavage of sphingosine-1 phosphate (S1P), into 2*E*-hexadecanal and phosphoethanolamine (91, 92). *SGPL1* is the major modulator of S1P signalling (93). Under normal physiological conditions, S1P is largely pro-proliferative, suppressing the pro-apoptotic actions of ceramide however loss of function mutations in *SGPL1* result in a pathological accumulation of S1P which studies have shown to be associated with induction of apoptosis.

In 2017, Prasad et al. (88) described loss of function human mutations in *SGPL1* and a novel syndrome characterised by primary adrenal insufficiency. Extra-adrenal phenotypic features included steroid-resistant nephrotic syndrome, hypothyroidism, ichthyosis, neurodevelopmental delay, hypogonadism and lymphopenia. Mass spectrometric analysis of plasma sphingolipids in one patient and heterozygous parents revealed elevated ceramides and S1P levels when compared to age and sex matched controls suggesting that the underlying multi-system pathology in these patients may be due to organ-specific cytosolic accumulation of sphingolipid intermediates. Correspondingly, Lovric et al. described a cohort of 7 families who were found on next generation sequencing to harbour 9 unique, recessive mutations in *SGPL1* (89). Several studies have subsequently corroborated these initial findings and expanded the phenotypic spectrum to include microcephaly, sensorineural deafness, and progressive neurological deterioration (94). A further neurological phenotype has been described in siblings bearing compound heterozygous loss of function mutations in *SGPL1*, involving axonal mononeuropathy giving rise to Charcot Marie Tooth-like disease (95).

*Sgpl1* ^-/-^ mice exhibit early postnatal mortality but those that survive demonstrate disrupted adrenal morphology. Adrenocortical zonation is disordered and cells of the zona fasciculata have reduced expression of steroidogenic enzymes and contain fewer lipid droplets when compared to wild type mice (88). Electron microscopy of *Sgpl1* ^-/-^ kidneys demonstrated foot process effacement and absent slit diaphragms (89). Tamoxifen-inducible *Sgpl1*-ablated (SPL^Flox/Flox^ Cre+) mice with partial lyase deficiency, interestingly, demonstrated glomerulopathy with progressive proteinuria and markedly increased intra-renal S1P levels (96). These mice additionally showed dermal irritation and hyperkeratosis whilst other phenotypic features of *Sgpl1* silencing were less evident suggesting that some degree of wild type activity may be protective against developing multi-systemic disease.

## 8 Conclusion

From initial descriptions of defects in the ACTH receptor to disorders of sphingolipid metabolism, the genomic landscape of FGD has dramatically transformed over the last three decades. Pending the discovery of novel genes implicated in pathogenesis of glucocorticoid deficient disorders, the likelihood of oligogenic inheritance is augmented in the diagnosis of unsolved FGD cases. One report suggests that digenic, tri-allelic inheritance of variants in both *STAR* and *CYP11A1* account for an isolated case of adrenal failure (97). Oligogenic heterozygosity is increasingly pertinent particularly in cases of haplotype ambiguity. Given the increasing accessibility of next generation sequencing techniques, the heritability of adrenal insufficiency-related phenotypes may be more easily uncovered allowing earlier patient intervention with significantly disease modulating impacts.

## Author contributions

AM: Writing – original draft, Writing – review & editing.
